# Relationship Between Morning Heart Rate Variability and Creatine Kinase Response During Intensified Training in Recreational Endurance Athletes

**DOI:** 10.3389/fphys.2018.01267

**Published:** 2018-09-13

**Authors:** Matthias Weippert, Martin Behrens, Anett Mau-Moeller, Sven Bruhn, Kristin Behrens

**Affiliations:** ^1^Institute of Sport Science, University of Rostock, Rostock, Germany; ^2^ISBA University of Cooperative Education, Schwerin, Germany

**Keywords:** training load, cycling, muscle damage, aerobic exercise, RMSSD, HF-Power, haemodilution, haematocrit

## Abstract

Specific physiological responses and their relationship were analyzed in 12 recreational endurance athletes (43.8 ± 7.9 years) during a period of intensified cycling training. Heart rate (HR), HR variability (HRV), serum creatine kinase (S-CK) and haematocrit (Hct) were measured in the mornings before (PRE) and following three consecutive days of intensified training (POST 1–3). Morning HR increased during this period (PRE: 52.2 ± 6.7 bpm, POST 1: 58.8 ± 7.0 bpm, POST 2: 58.5 ± 8.1 bpm, POST 3: 57.9 ± 7.2 bpm; *F*(3,33) = 11.182, *p* < 0.001, η_p_^2^ = 0.554). Parasympathetic HRV indices decreased from PRE to POST (*F*(3,33) ≥ 11.588, *p* < 0.001, η_p_^2^ ≥ 0.563), no effect was found for sympathetically modulated HRV (*F*(3,33) = 2.287, *p* = 0.101, η_p_^2^ = 0.203). Hct decreased (PRE: 49.9 ± 4.0%, POST 1: 46.5 ± 5.1%, POST 2: 45.5 ± 3.8%, POST 3: 43.2 ± 3.4%; *F*(3,33) = 11.909, *p* < 0.001, η_p_^2^ = 0.520) and S-CK increased during the training period (PRE: 90.0 ± 32.1 U/L, POST 1: 334.7 ± 487.6 U/L, POST 2: 260.1 ± 303.4 U/L, POST 3: 225.1 ± 258.8 U/L; *F*(3,33) = 3.996, *p* = 0.017, η_p_^2^ = 0.285). S-CK release was associated with HR (*r* = 0.453, *p* = 0.002, *n* = 44), RMSSD (*r* = −0.494, *p* = 0.001, *n* = 44) and HF-Power (*r* = −0.490, *p* = 0.001, *n* = 44). A period of intensified training was associated with haemodilution, parasympathetic withdrawal and S-CK-increase. Cardiac autonomic control at morning rest correlated with the S-CK-release; and thus, may serve as a practical mean to complementary monitor and prescribe training load in this population.

## Introduction

Regular physical activity is a key factor in healthy aging and is associated with many health benefits including reduced risk for cardiovascular diseases, cancer, and diabetes (Warburton et al., 2006; Shiroma and Lee, 2010). However, also detrimental effects of excessive endurance training have been recently documented. Especially, in aging athletes and/or athletes with cardiovascular risk factors or predisposed to cardiac abnormalities, excessive training loads may increase the risk of cardiovascular diseases (Eijsvogels et al., 2016). Further, excessive endurance exercise may lead to a transient loss in skeletal myocyte integrity contributing to muscle fatigue (Fitts, 1994). Muscle fatigue, in turn, may affect perceived effort (Pageaux, 2014) and thus pacing and completion of exercise as well as adherence to the subsequent training sessions (Ekkekakis, 2017). On the other hand, recent reports suggest that transient increases of metabolic (by-)products, such as lactate and reactive oxygen species, produced during physical exercise, may trigger positive adaptations (Warburton et al., 2006; Gomez-Cabrera et al., 2008; Brooks, 2009). Thus, the release of biochemical markers like troponins and creatine kinases do not only indicate a loss of cell integrity after acute (prolonged) exercise, but might stimulate favorable adaptations to exercise as well (Scharhag et al., 2006). Summarizing, the release of biomarkers such as creatine kinase might link short-term exercise responses, indices of fatigue and functional overreaching as well as beneficial adaptive processes. However, their sampling and analysis is costly and often impracticable, not only in non-professional sports. Thus, there is still a need for valid and practicable means to monitor and prescribe the amount and intensity of exercise, especially in periods of increased training load (Halson and Jeukendrup, 2004). Easy accessible indices of cardiac autonomic function, such as morning heart rate variability (HRV), maximal and submaximal heart rate (HR) or heart rate recovery have been successfully used to prescribe daily training load in moderately fit persons (Kiviniemi et al., 2007, 2010) and linked to states of functional (FOR) or non-functional (NFOR) overreaching (Buchheit, 2014). However, the relation between states of (functional) overreaching and autonomic indices are equivocal, e. g., due to different tools to quantify fatigue and training load, individual profiles of autonomic responses and methodological inconsistencies (Buchheit, 2014). A classification of FOR, even in endurance athletes of lower level, often bases on statistical calculations of intra-individual performance variability of world class track and field sprinters (Hopkins et al., 1999) – and a rather arbitrarily described duration of performance decrement (Ten Haaf et al., 2017). Considering the methodological difficulties inherently associated with this approach of FOR-definition in lower class endurance athletes, it seems to be obvious, that states of FOR may not be reflected in linear autonomic adjustments or other physiological markers. While periods of intensified training are considered an important aspect of performance development, it is also controversial whether any form of FOR is in fact beneficial for optimal performance improvements (Aubry et al., 2015).

Interestingly, studies dealing with short-term effects of intensified training on autonomic function, seromarker release, and hematological properties are predominantly limited to elite athletes. Furthermore, investigations often focused on single aspects of the bodily response and particular competitions (Siegel et al., 1981; O’Connor et al., 1991; Hedelin et al., 2000; Uusitalo et al., 2000; Saunders et al., 2004; Shave et al., 2005; Fortescue et al., 2007; Schumacher et al., 2008; Millet et al., 2011; Williams et al., 2011; Bogdanis et al., 2013; Gore et al., 2013). For example, a linear increase in plasma volume, with red cell volume being unchanged, has been well described during short endurance training periods (Convertino, 1991). Although cycling is one of the most popular aerobic exercises in recreational sports, less is known regarding the association of training load, creatine kinase release, morning autonomic control and perceived effort during a period of intensified cycling in *recreational* endurance athletes. As such increases in training volume and/or intensity typically occur in annual training camps, it is of greater interest to analyze, whether there is a link between autonomic control and other physiological indices of training load in this population. A link between morning HR and HRV with serum creatine kinase (S-CK) would provide a physiological rationale for the use of morning HR-derived measures to monitor and prescribe training in recreational endurance athletes. Thus, the aims of this observational field study were to (i) assess autonomic, haematocrit (Hct) and S-CK responses during a period of intensified training in male cyclists; and (ii) to elucidate, whether there is a correlation between autonomic responses and S-CK release during a period of intensified training in this population. It was hypothesized that an increase of training load leads to significant elevations of S-CK as well as morning HR and to decreases in Hct and parasympathetic HRV indices. We further supposed the S-CK release during the observational period being significantly correlated with indices of autonomic function at morning rest, i. e., an increased S-CK-release is associated with reduced parasympathetic HR control.

## Materials and Methods

### Participants

Based on the assumption of a strong effect (*f* = 0.40) of intensified training on morning HR, an α-error probability of 0.05 and a power of 0.80, an *a*-*priori* sample size calculation (G^∗^Power 3.1, Germany) indicated a total of *N* = 10 persons to be required. Considering a drop-out rate of 20%, 12 recreational male endurance athletes (age: 43.8 ± 7.9 years; body weight: 75.6 ± 11.4 kg; body height: 181.4 ± 7.0 cm) were personally invited and gave their written informed consent to take part in this study. The study was approved by the local ethics committee at the University of Rostock and was conducted in accordance with the Declaration of Helsinki. All subjects gave written informed consent. Weekly training load in the six weeks before the training camp was heterogeneous – as typically in such a sample – and achieved on average 96.5 ± 56.9 km of cycling and 14.9 ± 11.2 km of running. The number of training sessions per week ranged from one to a maximum of six. All participants were non-smokers. Participants with current or a history of cardiovascular or orthopedic diseases and current pharmacological treatment were excluded from this study. Further, only participants stating their ability and willingness to complete the proposed training schedule were included.

### Protocol

In this observational field study autonomic response, Hct and S-CK were assessed on four consecutive days during a period of intensified training. During the training period, participants followed their usual individual night sleep schedule. A minimum of 15 h was scheduled between consecutive training sessions to ensure sufficient periods of passive rest and night sleep. After awakening and emptying the urinary bladder subjects recorded their beat-to-beat intervals for 5 min in supine rest using a ECG-based breast belt system providing a sampling rate of 1 kHz (t6, Suunto^®^ Inc., Finland) (Weippert et al., 2010; Buchheit, 2014). HR measurements were followed by capillary blood drawings from the left earlobe to determine Hct and S-CK. Daily HR measurements and blood sampling were carried out at the same time of the morning after an overnight fast in an upright sitting position.

Measurements in the morning before the first training session served as the individual baseline (PRE). Post-measurements were carried out in the mornings following the training days (POST 1–3). Daily cycling distance, altitude difference, maximum temperature of the day and relative humidity differed between the training sessions and were: 20.6 ± 17.5 km, 20°C and 68% at PRE; 105 km, 1800 m, 26°C, and 42% for training day 1; 122 km, 1700 m, 20°C, and 50% for training day 2; and 80 km, 1900 m, 20°C, and 54% for training day 3, respectively. After each training session participants were asked to rate the global perceived effort (RPE) of the training using an adapted German version of a Borg-scale. Daily training load was estimated in arbitrary units (a.u.) using the product of training session time (in hours) and training intensity assessed by RPE (Foster, 1998). Following an ecological valid approach, participants followed their normal nutrition schedule and drank *ad libitum* during and after the training sessions. While participants refrained from caffeinated beverages ≥12 hrs prior the HR-measurements, their use was not restricted during the training sessions across the training days. No additional ergogenic substances were used by the athletes during the study period.

### Data Analyses

HRV analyses were carried out using the software Kubios HRV 2.0. (University of Kuopio, Finland). Average HR was calculated for 5 min. HRV spectral indices (model: autoregressive) and the natural-log of the root mean square of the sum of the squared differences of adjacent heartbeat intervals (lnRMSSD) were calculated from the detrended (method: Smoothn priors) 5-min beat-to-beat measurements. The natural log-transformed spectral power in the high frequency band (lnHF) from 0.15 to 0.4 Hz and the normalized power in the low frequency range (LF n.u.) from 0.04 to 0.15 Hz were analyzed. While the power in the HF-band reflects vagal efferent activity – similar to lnRMMSD –, the normalized LF power (LF n.u.) is considered to mirror the portion of sympathetic HR modulation (Task Force of the European Society of Cardiology, and the North American Society of Pacing, and Electrophysiology, 1996). Because beat-to-beat interval data of one participant could not be recorded with sufficient quality (artifact rate > 5%), HR and HRV data, as well as correlation analysis between HR-derived indices and S-CK are reported for 11 subjects only.

Serum creatine kinase and Hct were measured spectro photometrically (Vario II Photometer, Diaglobal, Germany) using commercially available detecting kits (CK 321, HCT 142, diaglobal, Germany). Hct was determined by a turbidimetric method. The S-CK assay bases on an enzymatic method, where S-CK is equivalent to the rate of NADPH formation that absorbs at 340 nm. All S-CK measurements were corrected for the individual Hct value. The catalytic concentration of S-CK is provided in U/L at 37°C (Schumann et al., 2002).

### Statistics

An analysis of variance for repeated measures (RM-ANOVA) and Bonferroni adjusted *post hoc* pair wise comparisons were conducted to test for significant training effects on the specific physiological responses. Since S-CK data violated the assumption of normal distribution, z-transformed values were used for the ANOVA. Standardized differences were used to evaluate the magnitudes of the PRE-POST differences of morning HR and HRV (Hopkins et al., 2009). Therefore, the alterations of HR and HRV were related to the smallest worthwhile change (SWC). An effect can be rated trivial if an individual change is within the SWC. SWC was calculated by 0.2 times of the between-athlete variation at PRE (Hopkins, 2004). Pearson’s correlation coefficient was used to assess the association between individually z-transformed morning HR and HRV with z-transformed S-CK. Thresholds of 0.1, 0.3, and 0.5 for small, moderate and large correlations were used according to Cohen (1988). Fisher’s exact significance was used to test for differences in training commitment in athletes with or without above normal S-CK. Data were analyzed using the SPSS statistical package 22.0 (SPSS Inc., Chicago, IL, United States) and statistical significance was accepted at *p*≤ 0.05.

## Results

Perceived effort was rated 16.4 ± 1.5, 15.3 ± 1.1, and 17.1 ± 1.0 for training days 1, 2, and 3, respectively. Training day had a significant effect (*F*(18,2) = 6.041, *p* = 0.010, η_p_^2^ = 0.402) with effort rated lowest for training day 2 (day 1 vs. day 2: *p* = 0.075, day 2 vs. day 3: *p* = 0.001). Calculated daily training load showed a different profile: 61.6 ± 5.2 a.u. for day 1, 69.1 ± 4.9 a.u. for day 2, and 62.4 ± 3.9 a.u. for day 3 (*F*(18,2) = 9.532, *p* = 0.002, η_p_^2^ = 0.514). Training load calculated for day 2 was significantly higher than for day 1 (*p* = 0.007) and day 3 (*p* = 0.001). No differences were found between day 1 and day 3.

Further, RM-ANOVA revealed a significant effect of time on Hct, with the highest value at PRE and the lowest value at the end of the training camp (POST 3) (**Table 1**). *Post hoc* analysis showed that Hct progressively decreased and differed significantly between all time points, except POST 1 and POST 2. The intensified training also significantly increased S-CK. Furthermore, at POST 1 S-CK values of three participants were markedly increased above the clinical cut-off of 200 U/L. Two of them reported stronger perceptions of fatigue and interrupted training on the second training. Fishers’s exact test revealed that the association between S-CK and training maintenance yielded statistical significance (*p* = 0.045).

**Table 1:** Mean ± standard deviation and repeated measures ANOVA statistics for morning haematocrit (Hct), serum creatine kinase (S-CK), heart rate (HR), natural log-transformed root mean square of the sum of the squared differences between adjacent inter-beat intervals (lnRMSSD), high frequency power (lnHFP), and normalized low frequency power (LF n.u.) of heart rate variability across the training period.

	PRE	POST 1	POST 2	POST 3	*F*	Sig.	Partial η^2^
Hct [%]	**49.9** ± 4.0	**46.5** ± 5.1^∗^	**45.5** ± 3.8^∗^	**43.2** ± 3.4^∗∗^††§	11.909	0.000	0.520
S-CK [U/L]	**90.0** ± 32.1	**334.7** ± 487.6	**260.1** ± 303.4^∗∗^	**225.1** ± 258.8^∗^	3.996	0.017	0.285
HR [bpm]	**52.2** ± 6.7	**58.8** ± 7.0^∗∗^	**58.5** ± 8.1^∗∗^	**57.9** ± 7.2^∗∗^	11.182	0.000	0.554
lnRMSSD [ms]	**3.8** ± 0.6	**3.4** ± 0.4^∗∗^	**3.4** ± 0.5^∗∗^	**3.4** ± 0.4^(∗)^	11.588	0.000	0.563
lnHFP [ms^2^]	**6.4**± 0.9	**5.6**± 1.0^∗∗^	**5.7**± 1.1^∗∗^	**5.7** ± 0.9^∗∗^	12.761	0.000	0.586
LF n.u.	**0.60** ± 0.23	**0.74** ± 0.13	**0.69** ± 0.20	**0.68** ± 0.21	2.287	0.101	0.203

*^(∗)∗^/^∗∗^ = different from PRE at a p-level of 0.071/ < 0.05/ < 0.01; †/†† = different from POST1 at a p-level < 0.05/ < 0.01; § = different from POST 2 at a p-level < 0.05.*

The effect of measurement time was also significant for HR and the vagally related HRV-indices lnRMSSD and lnHFP, while LF n.u. remained statistically unchanged (**Table 1**). The alterations of lnRMSSD and lnHFP from PRE to POST exceeded the SWC in all but one athlete.

**FIGURE 1:**
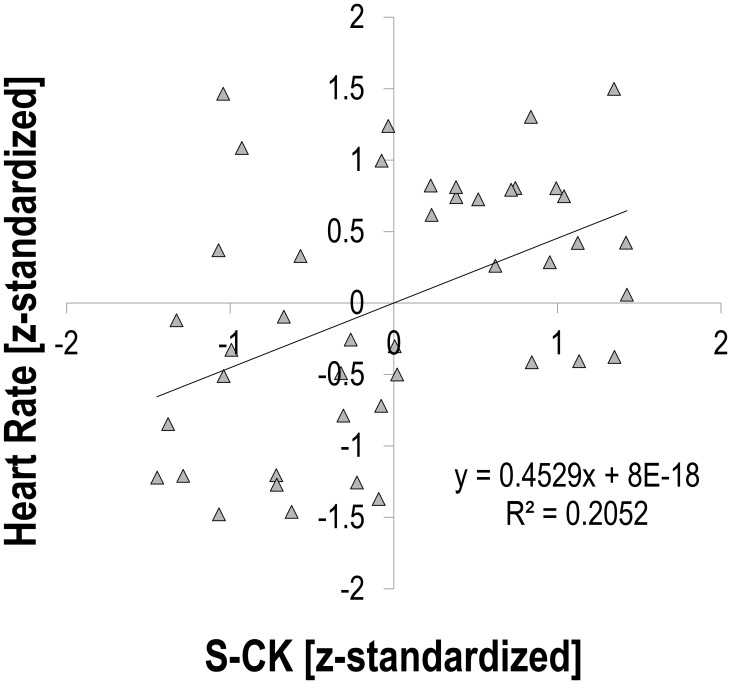
Correlation between heart rate and creatine kinase (S-CK).

**FIGURE 2:**
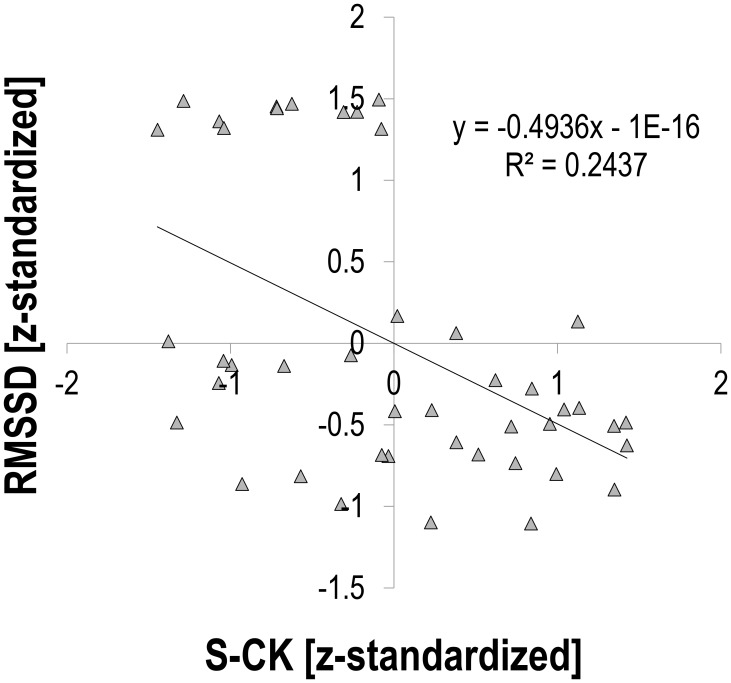
Correlation between HRV root mean square of the sum of the squared differences of adjacent heartbeat intervals (RMSSD) and creatine kinase (S-CK).

**FIGURE 3:**
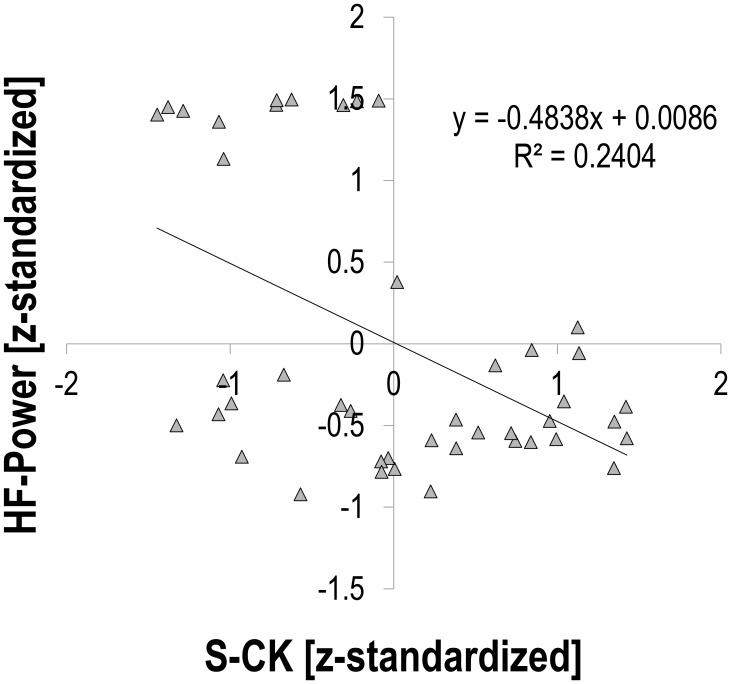
Correlation between HRV high frequency (0.15 – 0.4 Hz) power (HF-Power) and creatine kinase (S-CK).

There was a moderate relationship between S-CK and vagally mediated HRV indices. Pearson’s *r* for z-transformed S-CK vs. z-transformed HR was 0.453 (*p* = 0.002, *n* = 44, **Figure 1**), vs. z-transformed RMSSD −0.494 (*p* = 0.001, *n* = 44, **Figure 2**), and vs. z-transformed HF-Power −0.490 (*p* = 0.001, *n* = 44, **Figure 3**), respectively; while HRV sympathetic modulation index LF n.u. did not show any significant association with S-CK (*r* = −0.050 *p* = 0.750, *n* = 44). Pearson’s *r* for z-transformed HR with z-transformed RMSSD and HFP was −0.788 and −0.808 (*p* = 0.000, *n* = 44), respectively, while no association was found between HR and z-transformed LF n.u. (Pearsons’ *r* = −0.077, *p* = 0.624, *n* = 44).

## Discussion

In this observational field study training load, perceived effort, autonomic response (HRV indices), Hct and S-CK were assessed in recreational cyclists during a period of intensified training. The aim was to analyze alterations of and associations between these specific markers.

### Training Load and Perceived Effort

Calculated training load and session effort ratings showed a slightly different profile across the period of intensified training, with training load being highest and perceived effort lowest for training day 2.

### Biomarkers

In accordance with the training load profile, a significant increase of S-CK was first detected after the second day of the intensified training period – the day that significantly exhibited the highest calculated training load. This finding supports the use of S-CK as an objective marker for training load in recreational active athletes, since it seems to be associated to both, intensity and duration of exercise (Evans et al., 1998; Banfi et al., 2012). Generally, increases in S-CK may speak for detrimental effects of unaccustomed bouts of prolonged exercise on cellular integrity possibly due to increased oxidative stress (Ohta et al., 2009; Brancaccio et al., 2010; Banfi et al., 2012). Further, a loss in myocyte integrity may have increased muscle fatigue (Fitts, 1994) and might have led to adjustments in effort (Pageaux, 2014). This assumption would fit well to the finding of reduced perceived effort on training day 2. However, conclusions have to be drawn cautiously since the molecular mechanisms that result in CK release from muscle after mild exercise are still incompletely understood (Baird et al., 2012). Further, many other factors such as motivation or mental fatigue may impact on perceived effort and the adherence to exercise and training (Pageaux, 2014; Enoka and Duchateau, 2016; Ekkekakis, 2017; Van Cutsem et al., 2017; Schmit and Brisswalter, 2018).

The measured fall in Hct across the days might be due to the well-known exercise-induced hypervolemia (Convertino, 1991, 2007). Further, ambient temperatures during the study period were much warmer (around + 10 – 15°C) if compared to the weeks prior the camp. Thus, these temperatures – despite not being exceptionally high – might have further amplified the exercise-induced heat production in our “unacclimatized” study participants and contributed to the plasma volume expansion (Convertino et al., 1980; Convertino, 1991, 2007; Lorenzo et al., 2010; Buchheit et al., 2011). In this respect, assessment of sweating rate might have helped to further elucidate the underlying contributions; however, unconfounded assessment of body mass and fluid intake monitoring were not implementable in this setting.

### Autonomic Control Measures

The increase of the morning HR at all POST-days compared to the PRE-measurements implies that a resting period of ≥ 15 h, including night sleep, is insufficient to permit complete recovery from an unaccustomed bout of cycling exercise. The increase in HR was about 10% and thus exceeded the SWC in this sample (Buchheit, 2014). Additionally, it has to be considered that an increase in plasma volume across the training period might have reduced the increase in morning HR and decrease in morning HRV, respectively (Buchheit et al., 2011). The alteration of lnHFP (about 12% reduction from PRE to POST) reflects a reduction of vagal HR modulation on all POST days. The effect of intensified training on morning HR, lnRMSSD and lnHFP is not only statistically significant but most likely substantial, using the SWC criteria given by Hopkins (2006). Furthermore, a substantial effect on LF n.u. was very likely at POST 1, likely at POST 2, and possible at POST 3. Results might also speak for a distinct autonomic recovery pattern in response to intensified training, with the vagal rebound taking longer compared to the sympathetic withdrawal. However, it has to be considered that (i) the change in LF n.u., was statistically insignificant and (ii) LF-HRV may rather reflect baroreflex mediated autonomic outflow than sympathetic tone (Goldstein et al., 2011). Further, considering the strong correlation between HR and lnRMSSD and lnHFP, respectively, the parasympathetic branch of the autonomic nervous system might not be the exclusive, but the main contributor to the increase of morning HR during this period of intensified training. Despite equivocal results regarding the potential of HR and HRV in detecting states of FOR or NFOR and training load in elite athletes (Bosquet et al., 2008; Buchheit, 2014; Plews et al., 2014; Coates et al., 2018), our data support the view that morning HR and HRV can reflect larger alterations in training load in recreational athletes.

### Associations Between S-CK, HR, and HRV

Correlation analysis for S-CK and HRV showed that individual changes of morning-HR and -HRV can explain up to 25% of the change in S-CK, pointing to the potential of these measures to complementary objectify and monitor training status. Furthermore, a traditional seromarker of myocyte injury and training load was associated with alterations of HR-derived indices of autonomic control, a finding that has been previously reported by only a few investigators (Buchheit et al., 2011).

### Limitations

Because we did not assess the physiological alterations following the training camp after resuming to the “normal” routine, this study is limited to relative short-term responses to training volume increases. Further, no “classical” index of (functional) overreaching, such as transient performance decrements, has been assessed. In this respect it is of note that it is currently debated, whether any form of overreaching is, in fact, beneficial for adaptations and performance (Aubry et al., 2014). Further, only training volume but not intensity were assessed for the 6 weeks prior to the study period. However, none of the athletes reported subjective signs of FOR or NFOR and also S-CK as well as HR were in a normal range at the beginning of the intensified training period.

## Conclusion

From these analyses, we cautiously conclude that morning HR and HRV may serve as practical, complementary measures to monitor functional status in recreational endurance athletes during periods of intensified training. Since the profile of morning HR and HRV was associated with increases in training load and the levels of a seromarker of muscle strain, a decrease in HRV/ increase in HR may be indicative of non-sufficient recovery from unaccustomed bouts of endurance exercise in these athletes.

## Author Contributions

MW and KB designed this study. MW collected, analyzed, and interpreted the data. MW and MB drafted the manuscript, all authors revised the manuscript and approved the final version to be published.

## Conflict of Interest Statement

The authors declare that the research was conducted in the absence of any commercial or financial relationships that could be construed as a potential conflict of interest.
